# Reliability Analysis of Bond Behaviour of CFRP–Concrete Interface under Wet–Dry Cycles

**DOI:** 10.3390/ma11050741

**Published:** 2018-05-07

**Authors:** Hongjun Liang, Shan Li, Yiyan Lu, Ting Yang

**Affiliations:** The School of Civil Engineering, Wuhan University, Wuhan 430000, China; hongjunliang8@163.com (H.L.); yylu901@163.com (Y.L.); yangt329@163.com (T.Y.)

**Keywords:** reliability analysis, FRP-concrete, bond behaviour, wet–dry cycles, sustained loading

## Abstract

Effective bonding between adherents plays a key role in retrofitting concrete structures in civil engineering using fibre-reinforced polymers (FRPs). To ensure structural safety, it is critical to develop design codes, which account for uncertainties of materials, the environment, and load, to estimate bond behaviour under long-term exposure to harsh environments. Therefore, a reliability analysis was performed to study the bond behaviour of FRP–concrete interface under wet–dry cycles and sustained loading. Thirty double-lap, shear-bonded carbon FRP (CFRP)–concrete composite specimens were tested after wet–dry cycles and sustained loading exposure. The fracture energy *G*_f_ of the bond behavior between CFRP and concrete was directly obtained from the measured local bond-slip curves. Five widely used test methods were adopted to verify the possible distribution types of *G*_f_. Based on the best fit distribution of *G*_f_, a reliability index *β* was then calculated for the specimens. The effects of wet–dry exposure and sustained loading on *β* were analysed separately. The effects of the mean and standard deviation of the load on *β* were compared. It was found that the mean had a greater impact on reliability than the standard deviation, but neither changed the regulation of the exponential reduction of *β* with increasing wet–dry cycle time. Their impact was significant for a small number of wet–dry cycles but insignificant for more than 4000 wet–dry cycles.

## 1. Introduction

Externally bonded fibre-reinforced polymer (FRP) composites have been shown to be an effective strengthening method for existing engineering structures due to its excellent tensile strength, light weight, corrosion resistance, and easy tailoring [1,2]. The performance of this method depends on adequate bond strength between the FRP and the concrete, which ensures the stress transmission between and coordination of FRP and concrete [3]. Therefore, numerous studies have been conducted on the bond mechanism of the FRP-to-concrete interface, including experimental studies [4,5,6,7], theoretical analysis [8,9,10,11], and finite-element simulations [12,13]. The vast majority of these studies have used fracture energy, *G*_f_, as an indicator for evaluating the FRP–concrete interfacial bond strength. Several empirical models [14,15,16,17,18,19,20] have been proposed for predicting the *G*_f_ based directly on the regression of test data or theoretical models based on fracture mechanics.

However, many uncertainties exist in the parameters of these models, such as concrete tensile strength, FRP stiffness, the width ratios of FRP, and concrete and the shear stiffness of the adhesive layer. Variation in mechanical properties and the geometry of FRP composites and adhesive layer can cause significant deviations from the intended design performance of an FRP rehabilitation, increase the probability of failure, and reduce the safety of the structure [2]. Kaiser and Karbhari [21] identified sources of uncertainty in wet layup composites such as wrinkled or broken fabric strands, the use of incorrect or tainted resin mixtures, poor site preparation, and the misplacement of fabric during layering. Similarly, Atadero et al. [22] claimed that wet layup materials can show greater variability than pultruded laminates and steel plates. A coefficient of variation (COV) of 0.10 should be assumed for wet layup when quality control is better than average, a COV of 0.15 when quality control is average, and a COV of 0.20 when quality control is below average. In addition, FRP composite structures in actual service conditions are often exposed to harsh environments (e.g., marine environments with penetrative moisture, acid rain erosion, excessive heating, and sustained load coupling) [23]. These harsh environments further increase the randomness of the uncertain parameters regarding the long-term performance of FRP composites [2,22]. As a result, the accuracy of the limit state format falls and unconservative estimates may even arise in the design process, further increasing the risk that strengthening will fail. Karbhari et al. [24] demonstrated that existing methods are unable to accurately account for the effects of severe long-term environmental exposure on FRP systems. The use of safety factors or partial safety factors prescribed by ACI-440 [25] and TR-55 [26] does not take into account the effects of variation on overall reliability and can result in a false sense of reliability. Therefore, such standards and guidelines must address uncertainties of mechanical properties and geometry of FRP composites, uncertainties in existing structures, and long-term performance under exposure to multiple harsh environments [27,28,29,30].

A probabilistic method was often used by many researchers to analyse the behaviour of FRP-strengthened concrete structures, which can account for those uncertainties associated with design parameters and quantify the probability of failure [31]. Val [32] evaluated the reliability of FRP-confined reinforced concrete columns using FORM-based technique. Uncertainties associated with material (i.e., concrete and FRP composite) strength, models predicting the ductility of FRP-confined concrete, and dead and live loads were taken into account. On the basis of reliability analysis, tentative suggestions were recommended to modify the strength reduction factor for the design of FRP-confined columns. Atadero et al. [30] investigated and assessed the variability accrued through the use of wet-bonded FRP to strengthen the decks of a bridge. All of the slabs were strengthened sufficiently that their reliability exceeded the requirements of the AASHTO LRFD Bridge Design Specifications [33], but the degradation of the FRP materials over time and the potential statistical effects of this degradation were not considered. Wieghaus et al. [34] investigated the effects of different levels of uncertainty in existing reinforced concrete (RC) structures in addition to the variability of materials on the reliability of repairs designed with ACI 440.2R-08 recommendations, and their importance was demonstrated when designing FRP repair for deteriorated RC members. Wang et al. [35] developed a probability-based limit state standard for the design and evaluation of RC structures strengthened by externally bonded CFRP composites. They found that a separate resistance factor of 0.75 should be applied to the nominal strength of the CFRP to achieve a reliability index of approximately 3.0, and that a partial factor should be applied separately to each strength variable if the strength is represented by a sum of random variables. However, their studies of resistance criteria take neither environmental factors nor structural deterioration into account.

In sum, probabilistic modelling has been adopted to account for the uncertainties of materials, existing structures, and long-term performance under multiple harsh-environments exposure. However, few studies were conducted on the reliability analysis of the bond behavior of FRP-concrete's interface under long-term environmental exposure. Therefore, the goal of this paper is to demonstrate the use of probabilistic modelling to evaluate long-term performance based on a series of double-lap pullout test results. The fracture energy *G*_f_ of the bond strength between FRP and concrete was directly obtained from measured local bond-slip curves. The possible distribution types of *G*_f_ and the reliability index *β* of the specimens were then calculated. Subsequently, a detailed investigation was conducted of the effects of wet–dry cycles and the mean and standard deviation of sustained loads on *β*.

## 2. Experimental Programme

**Figure 1:**
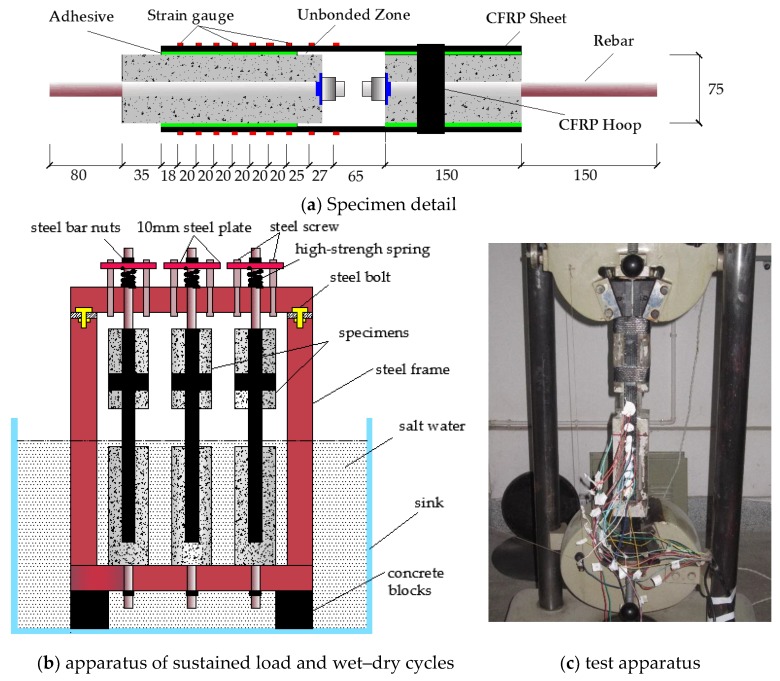
Details of specimens and apparatus.

The details of the experimental programme can be found in our previously published article [3]. Here, only a brief description is provided to ensure the completeness of the paper. Thirty CFRP–concrete composite specimens were designed and tested as shown in Table 1. Each composite specimen consisted of two FRP strips and two concrete prisms, as shown in Figure 1a. The dimensions of the two concrete prisms were 75 × 75 × 220 mm and 75 × 75 × 150 mm, respectively. To simulate the actual mechanical situation of the CFRP strengthened concrete structures, sustained loads were applied through a specially designed and self-made frame, as shown in Figure 1b. After applying the sustained loads, some of the specimens were fully immersed in a sink filled with 5% NaCl solution for 12 h and then subjected to accelerative blow-drying for another 12 h per cycle to simulate tidal changes. Once the predetermined exposure cycles had been completed, the coupons were placed in the lab to air dry. A total of nine high precision foil strain gauges, including two strain gauges in unbonded zone and seven strain gauges in the bonded zone, were then attached to the FRP strip to record the tensile load and monitor the strain variation along the FRP strips during the loading process. The length and the width of the active gage are 3.0 mm and 2.2 mm, respectively. The center-to-center distance of the adjacent strain gauge in the bonded zone is 20 mm, as shown in Figure 1a. The CFRP–concrete composite specimens were tested according to ASTM D3039, as shown Figure 1c. All of the data were recorded by an electronic acquisition system. The average concrete strength (*f*_cu_), Young’s modulus, and the ultimate strength of the CFRP are listed in Table 1.

## 3. Interfacial Fracture Energy, *G*_f_

The interfacial fracture energy, *G*_f_, is one of the most important indicators used to characterise FRP–concrete interfacial bond behaviour. The above experimental results indicated that the failure mode of the control specimens (CC-CON) was debonding in concrete with much concrete becoming attached to the CFRP strip. As this outcome is caused by insufficient tensile strength *f*_ct_ of concrete, so *f*_ct_ was the key parameter of many common models for predicting *G*_f_ of CFRP–concrete composite specimens. However, the strength of the adhesive decreased and *f*_ct_ increased continuously with an increase in wet–dry cycle time. The failure position of the specimens after wet–dry cycles and sustained loading ageing transferred from the concrete to the adhesive–concrete interface, and with less and less concrete attached to the CFRP strip. Therefore, the models used to predict *G*_f_ under normal circumstances could not be used to calculate the *G*_f_ of composite specimens under long-term environmental exposure.

**Figure 2:**
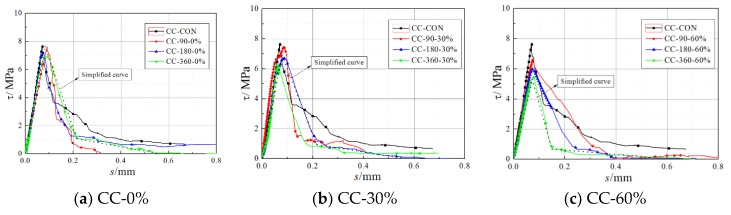
Local bond-slip curve of the specimens.

In this study, *G*_f_ was directly obtained by measuring the area underneath of the local bond-slip curves. The average shear stress of one side between micro-units *dx* and the integral relative slip can be calculated by Equations (1) and (2), respectively.

(1) $$\tau_{x}=E_{f}t_{f}(\varepsilon_{i}-\varepsilon_{i-1})/dx$$

(2) $$s_{i}=\frac{\Delta x}{2}(\varepsilon_{0}+2\sum_{j=1}^{i-1}\varepsilon_{j}+\varepsilon_{i})$$

where *E*_f_ and *t*_f_ are the elastic modulus and the thickness of FRP strip, respectively. *ε_i_*_+1_ and *ε_i_* are two adjacent strain readings at positions *i* + 1 and *i*, respectively. *dx* is the distance between the two adjacent gauge positions. Proceeding in this way for all of the gauge positions, the shear stress distribution along the bonded joint could be obtained and then integrated to get the shear stress of the overall FRP. The obtained local bond-slip curves of the specimens are shown in Figure 2.

The mean and coefficients of variation (COVs) of *G*_f_ after environmental exposure are listed in Table 1. As shown in the table, the mean of *G*_f_ decreased as wet–dry cycle time increased. The mean of the *G*_f_ of the CFRP–concrete composite specimens decreased by an average of 5.71%, 8.70%, and 13.09% after 90 d, 180 d, and 360 d, respectively, of wet–dry cycles exposure. Under 30% sustained loading, the reduction in *G*_f_ increased to 9.47%, 13.44%, and 17.62%, respectively. Under 60% sustained loading, this reduction increased further to 16.50%, 17.69%, and 28.62%, respectively. In addition, the COVs of the *G*_f_ of the specimens subjected to environmental exposure were significantly greater than those of the control specimens. The COV of CC-360-60% was 141% larger than that of CC-CON. Wet–dry cycles and sustained loading ageing were thus found not only to reduce the mean of *G*_f_ of FRP–concrete interface, but also to increase the dispersion of *G*_f_.

## 4. Discussion of Fracture Energy, *G*_f_

Recently, the parameter uncertainties in the bond resistance of CFRP–concrete composite specimens, such as concrete compressive strength, concrete modulus, FRP strength, FRP modulus, and FRP thickness, have been explored and found to fit Normal, Lognormal, Weibull, or Gamma distribution [24,32,34,36]. However, few studies have been conducted on the type of statistical distribution that best fits *G*_f_. Therefore, the fit of these four typical distribution types to the *G*_f_ data was investigated. In order to determine which distribution was the best fit for the data, five widely used test methods, the Shapiro–Wilk (S–W) test, the Kolmogorov–Smirnov (K–S) test, the Cramér–von Mises (C–M) test, the Anderson–Darling (A–D) test and the Chi-squared (χ^2^) test, were adopted.

The *G*_f_ of the specimens without environmental exposure, under wet–dry cycles exposure only, under wet–dry cycles exposure and 30% sustained loading, and under wet–dry cycles exposure and 60% sustained loading exposure, respectively, were considered to form one sample. The probability distributions of the four samples were analysed using the statistical software package SAS (Statistical Analysis System) and the goodness of fit (*p*), as listed in Table 2a–d. The size of *p* indicates the degree of coincidence for each probability distribution type. A larger *p* value indicates a greater probability that the sample will obey the given distribution. In this paper, *α* = 0.1 was used as the significance level, so when *p* > 0.1, the sample was considered to fit the distribution in question. As shown in Table 2a, the *p* values of the specimens without environmental exposure under the five test methods were all greater than 0.1 for the normal distribution. For the lognormal distribution, the *p* values were greater than 0.1 under all of the test methods except the χ^2^ method. However, the *p* values for the lognormal distribution were larger than those for the normal distribution under all four test methods except the χ^2^ method. The *p* values for the Weibull distribution were also lower than those for the lognormal distribution in most cases. The results for the gamma distribution could not be verified by most of the test methods due to the small sample size. So, in general, the lognormal distribution was considered to be the best fitting distribution type.

As shown in Table 2b, the *p* values of the specimens under wet–dry exposure only were larger than 0.1 under every test method. Therefore, the *G*_f_ of the specimens subjected to only wet–dry exposure followed the four tested distributions. However, the *p* values for the lognormal and gamma distributions were larger than those for the normal and Weibull distributions. So, the lognormal and gamma distributions were more appropriate to the *G*_f_ of the specimens subjected only to wet–dry exposure, with similar goodness-of-fit values.

As shown in Table 2c, the *G*_f_ of the specimens subjected only to wet–dry cycles and 30% sustained loads was considered to follow all four tested distributions, due to the large values of *p*. Thereinto, the lognormal distribution was again found to be the best fit for the *G*_f_ of the specimens subjected to wet–dry cycles and 30% sustained loads because it had a larger *p* value than the other three distributions under all five test methods (except that in the K–S test, the *p* value for lognormal distribution was less than that for the gamma distribution).

Table 2d shows that the *G*_f_ of the specimens subjected to wet–dry cycles and 60% sustained loads followed the four distributions fairly closely, as the *p* values were far larger than 0.1 in all cases. Again, the *p* values for the lognormal and gamma distributions were larger than those for the normal and Weibull distributions. Although that the *p* values for the gamma distribution were larger than those for the lognormal distribution under the K–S and χ^2^ methods, a lognormal distribution was considered to be the optimal probability distribution of the *G*_f_ of the specimens subjected to wet–dry cycles and 60% sustained loads. This was because that the *p* value for the gamma distribution could not be calculated using the S–W test and the distribution type under other circumstances is preferably uniform to facilitate calculation. Another observation was that the *p* values of the specimens subjected to both wet–dry cycles and sustained loads were significantly larger than those of the specimens not exposed to wet–dry cycles. This may be due to the larger COV induced by wet–dry exposure.

**Figure 3:**
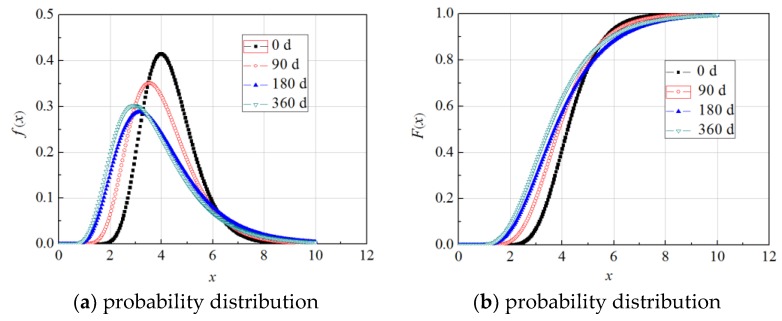
The probability distribution and cumulative distribution of typical specimens.

In sum, for the specimens not exposed to wet–dry cycles and the specimens subjected to both wet–dry cycles and sustained loads, the optimal probability distribution of the *G*_f_ of FRP–concrete bond strength was a lognormal distribution. Therefore, neither wet–dry exposure nor sustained loading changed the type of probability distribution of the *G*_f_ of FRP–concrete bond strength. Figure 3 shows the probability distribution and cumulative distribution of typical specimens.

## 5. Reliability Index of *G*_f_

To simultaneously consider accuracy and simplicity, the probability distribution of the *G*_f_ of FRP–concrete interfacial was considered to fit a lognormal distribution. The limit state function or performance was represented by the following equation:

(3) Z=g(R,S)=R−S=0

where *R* is the random resistance and *S* is the load effect.

The limit state equation for the degradation of *G*_f_ can be expressed as follows:

(4) $$Z=g(G_{fn}^{i},G_{fc})=G_{fn}^{i}(N)-G_{fc}(N)=0$$

where $G_{fn}^{i}(N)$ and $G_{fc}(N)$ are the resistance and load effect after *N* times wet–dry cycles respectively, and both obey a lognormal distribution. *i* was taken as 1, 2, and 3 for cases of unloading, 30% sustained loading, and 60% sustained loading, respectively. A simple transformation was undertaken as follows:

(5) $$\left\{ \begin{array}{l} G_{1}^{i}(N)=\ln G_{fn}^{i}(N)\\ G_{2}(N)=\ln G_{fc}(N) \end{array}\right.$$

where $G_{1}^{i}(N)$ and $G_{2}(N)$ obey a normal distribution.

Then Equation (3) can be converted to:

(6) $$Z=g(G_{1}^{i},G_{2})=e^{G_{1}^{i}}-e^{G_{2}}=0$$

In this study, the JC method was adopted to solve the reliability index (*β*). The direction cosine of the two coordinate vectors can be expressed as follows:

(7) $$\left\{ \begin{array}{c} \cos\theta_{{}_{1}}^{i}(N)=\frac{{-\frac{\partial g}{\partial G_{{}_{1}}^{i}(N)}|}_{P^{*}}\cdot \sigma_{{}_{1}}^{i}(N)}{\sqrt{\left[{(\frac{\partial g}{\partial G_{{}_{1}}^{i}(N)}|}_{P^{*}}\cdot \sigma_{{}_{1}}^{i}(N\right){)}^{2}+\left({\frac{\partial g}{\partial G_{2}(N)}|}_{P^{*}}\cdot \sigma_{2}(N\right){)}^{2}]}}=\frac{-e^{{\left(G_{1}^{i}(N)\right)}^{*}}\cdot \sigma_{{}_{1}}^{i}(N)}{\sqrt{\left(-e^{{\left(G_{1}^{i}(N)\right)}^{*}}\cdot \sigma_{{}_{1}}^{i}(N\right){)}^{2}+\sigma_{{}_{2}}^{2}(N)}}\\ \cos\theta_{2}^{i}(N)=\frac{{-\frac{\partial g}{\partial G_{2}(N)}|}_{P^{*}}\cdot \sigma_{2}(N)}{\sqrt{\left[{(\frac{\partial g}{\partial G_{{}_{1}}^{i}(N)}|}_{P^{*}}\cdot \sigma_{{}_{1}}^{i}(N\right){)}^{2}+\left({\frac{\partial g}{\partial G_{2}(N)}|}_{P^{*}}\cdot \sigma_{2}(N\right){)}^{2}]}}=\frac{\sigma_{2}(N)}{\sqrt{\left(-e^{{\left(G_{1}^{i}(N)\right)}^{*}}\cdot \sigma_{{}_{1}}^{i}(N\right){)}^{2}+\sigma_{{}_{2}}^{2}(N)}} \end{array}\right.$$

where $\sigma_{1}^{i}(N)$ is the standard deviation of $G_{1}^{i}(N)$ and $\sigma_{2}(N)$ is the standard deviation of $G_{2}(N)$.

The coordinates of the check point in the original coordinate system were as follows:

(8) $$\left\{ \begin{array}{l} {\left(G_{1}^{i}\right)}^{*}=m_{1}^{i}(N)+\beta_{i}\sigma_{1}^{i}(N)\cos\theta_{1}^{i}(N)\\ {\left(G_{2}^{i}\right)}^{*}=m_{2}(N)+\beta_{i}\sigma_{2}(N)\cos\theta_{2}^{i}(N) \end{array}\right.$$

where $m_{1}^{i}(N)$ is the mean of $G_{1}^{i}(N)$ and $m_{2}(N)$ is the mean of $G_{2}(N)$.

Substituting the coordinates of the check point into Equation (4) gave:

(9) $$e^{{\left(G_{1}^{i}\right)}^{*}}-e^{{\left(G_{2}^{i}\right)}^{*}}=0$$

The *β* of the FRP–concrete interface was obtained iteratively using Equations (5)–(7) as follows:

(10) $$\beta =\frac{m_{1}^{i}(N)-m_{2}(N)}{\sqrt{{\left(-{\sigma_{1}}^{i}(N)\right)}^{2}+{\sigma_{c}}^{2}(N)}}$$

The corresponding interface degradation probability was calculated by P=Φ(−β).

The mean $m_{2}(N)$ and standard deviation $\sigma_{2}(N)$ of the resistance were assumed to be constant and equal to the test value. $m_{1}^{i}(N)$ and $\sigma_{1}^{i}(N)$ were regressed from the fit of the experimental results as shown in Equations (11)–(16):

(11) $$m_{1}^{1}(N)=-0.0009N+0.3552$$

(12) $$m_{1}^{2}(N)=-0.0010N+0.3451$$

(13) $$m_{1}^{3}(N)=-0.0012N+0.3142$$

(14) $$\sigma_{1}^{1}(N)=0.0005N+0.1902$$

(15) $$\sigma_{1}^{2}(N)=0.0005N+0.2050$$

(16) $$\sigma_{1}^{3}(N)=0.0005N+0.2153$$

**Figure 4:**
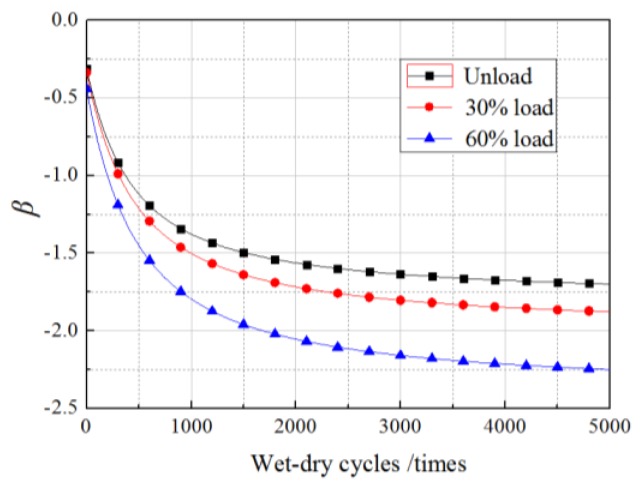
Change of *β* with wet–dry cycles.

**Figure 5:**
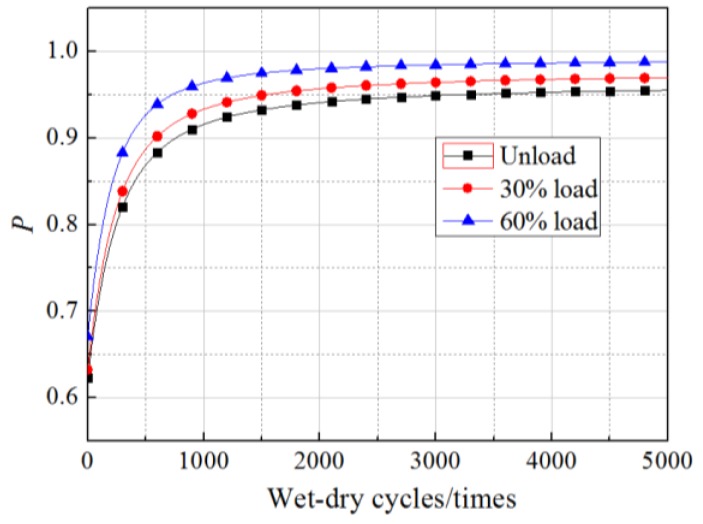
Change of *P* with wet–dry cycles.

The *R*^2^ values obtained from fitting Equations (11)–(16) were 0.8135, 0.8437, 0.7911, 0.8644, 0.8779, and 0.8035, respectively, which illustrated the regression equations were relatively accurate. The values of *N* in the equations were within the scope of the experimental research, ranging from 0 to 360 times. However, in the follow-up analysis of the *β* of *G*_f_, the equations were extended to predict the bond action beyond 360 times, but the precision of the prediction requires further examination by experimental data. Setting the load to the experimental values and substituting Equations (11)–(16) into Equation (10), *β* in cases of unloading, 30% loading, and 60% loading in our experimental conditions were predicted along with wet–dry cycle time. The results are shown in Figure 4 and Figure 5.

As shown in the figures, for all the specimens under a sustained load and those without sustained loading, the change in the *β* of *G*_f_ with wet–dry cycle time were divided into two stages, comprising a fast turn-off and a slow turn-off, where the overall trend obeyed a logarithmic function. Taking the unloaded case as an example, the *β* of *G*_f_ decreased rapidly from −0.2 to −1.35 after 1000 wet–dry cycles. In contrast, during the 1000 wet–dry cycles to 4000 wet–dry cycles, the *β* of *G*_f_ decreased slowly from −1.35 to −1.55. Accordingly, the failure probability increased rapidly from 0.6 to 0.95 after 1000 wet–dry cycles and increased slowly from 0.95 to 0.97 from the 1000 wet–dry cycle times to the 4000 wet–dry cycles.

Under the dual action of wet–dry exposure and sustained loading, the *β* (or failure probability) decreased (or increased) markedly as sustained loading levels increased. The influence of the sustained loads was exaggerated as the number of wet–dry cycles increased. After 200 times of wet–dry cycles, *β* had decreased by 6% and 15% for specimens subjected to 30% and 60% sustained loads, respectively, relative to its values for unloaded specimens. After 2000 wet–dry cycles, the equivalent reductions reached up to 12% and 24%, respectively.

## 6. Effects of Load Distribution on Reliability of *G*_f_

In this section, to provide a theoretical basis for determining a reasonable design value, the effects of the mean value and standard deviation of the load on the *β* of FRP–concrete bond behaviour are analysed in detail.

### 6.1. Effects of Mean Values of Load

**Figure 6:**
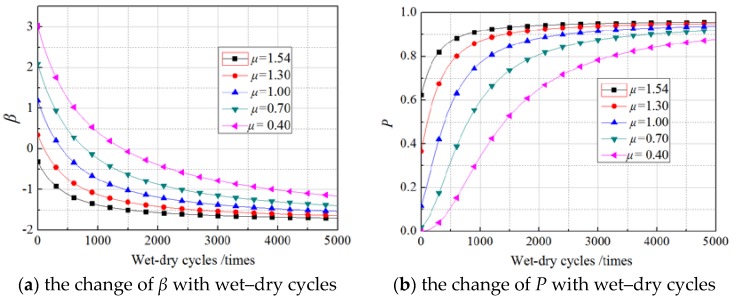
The effects of mean of the load on the nonsustained loading specimens.

**Figure 7:**
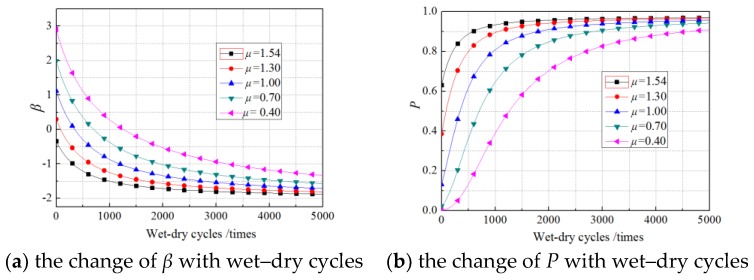
The effects of mean of the load on the 30% sustained loading specimens.

**Figure 8:**
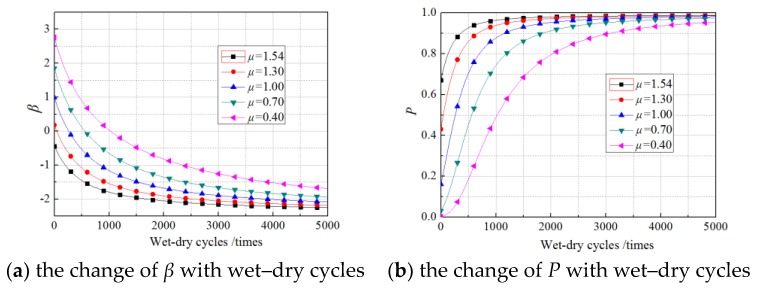
The effects of mean of the load on the 60% sustained loading specimens.

The mean values of the load were determined according to the design values and the experimental values of *G*_f_. The design values were calculated by several common models proposed in the literature [10,16,18,19,20]. The range of calculated values of *G*_f_ was 0.38 N/mm–1.10 N/mm. Given the experimental value of 1.54 N/mm, the mean values of the load in this study were set as 1.54 N/mm, 1.30 N/mm, 1.00 N/mm, 0.70 N/mm, and 0.40 N/mm, and the corresponding *β* and deterioration probability of *G*_f_ were calculated by the formula in Section 5. Figure 6, Figure 7 and Figure 8 show the calculated results, where the standard deviation of *G*_f_ was set equal to the experimental value (0.2522 N/mm).

As displayed in Figure 6, Figure 7 and Figure 8, for different mean values, the *β* of *G*_f_ showed an exponential decrease as the number of wet–dry cycles increased, while the failure probability increased exponentially with increasing wet–dry cycle time. The rate of reduction of *β* (rate of increase of failure probability) was higher in the early period of wet–dry exposure but remained changeless beyond a certain wet–dry cycle exposure. For lower mean load values, the *β* is clearly greater than that of a higher mean load at the beginning of the wet–dry cycle. For example, the *β* of a specimen under a load with a mean of 0.40 N/mm is about 3.02 at the first wet–dry cycle, whereas the *β* of a specimen under a load with a mean of 1.00 N/mm is only 1.19 at the first wet–dry cycle. However, after 4000 wet–dry cycles, the difference in *β* of specimens under loads with different mean was very minor and their failure probability approaches 1.0. This is mainly due to the sharply degraded bond behavior under the continuous erosion of wet–dry cycles and sustained loading. For cases of 0, 30%, 60% sustained loads, the influence rules of mean values of the load were highly similar.

### 6.2. Effects of Standard Deviation of the Load

**Figure 9:**
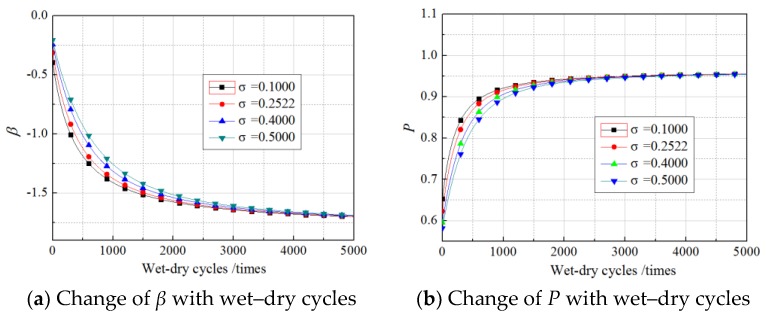
The effects of standard deviations of the load on the nonsustained loading specimens.

**Figure 10:**
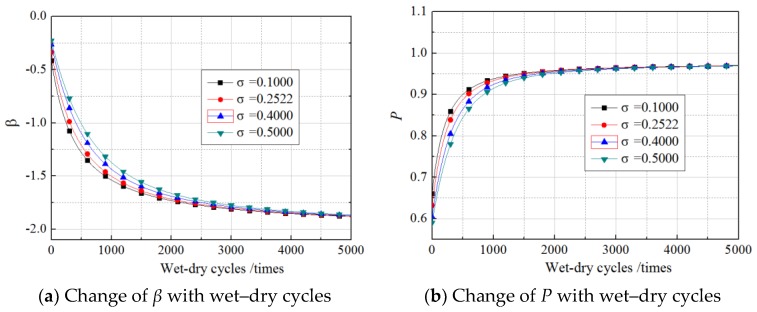
The effects of standard coefficients of the load on the 30% sustained loading specimens.

**Figure 11:**
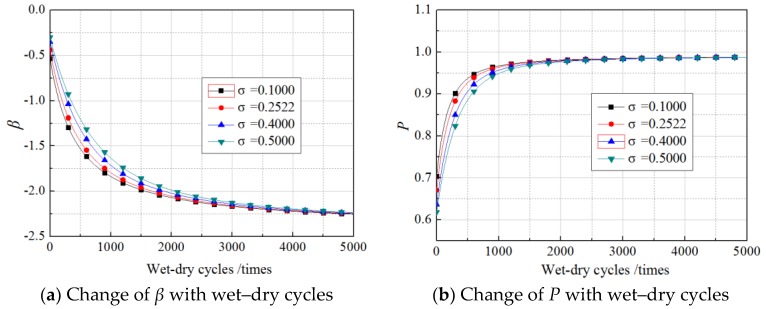
The effects of standard coefficients of the load on the 60% sustained loading specimens.

Due to the lack of specific formula and regulations governing the standard deviation of the load, the standard deviations of the load were set as 0.1000 N/mm, 0.2522 N/mm, 0.4000 N/mm, and 0.5000 N/mm according to the experimental results. The *β* values and failure probability were calculated as explained in Section 5, and the results are shown in Figure 9, Figure 10 and Figure 11. The curves were obtained using the experimentally obtained average value of the load, 1.54 N/mm.

As shown in Figure 9, Figure 10 and Figure 11, in every loading case, the standard deviations of the load did not change the *β* or the exponential growth of failure probability with increasing wet–dry cycle times. However, in the first stage with rapid changes, a larger standard deviation corresponded to a lower *β* and a higher failure probability, and the differences induced by different standard deviations of the load was smaller than those induced by different mean values of the load. After 2000 wet–dry cycles, the differences induced by different standard deviations of the load were negligible.

In sum, neither the mean values nor the standard deviations of the load changed the regulation of *β*, which showed exponential growth with increasing wet–dry cycle times, but both had a significant influence on the *β* values. This influence was significant in the stage of a small number of wet–dry cycles but insignificant for more than 4000 times of wet–dry cycles.

## 7. Conclusions

Thirty improved double-lap shear bond CFRP–concrete composite specimens were tested after experiencing a maximum of 360 days of wet–dry cycles and sustained loading to a maximum of 60% of the ultimate load. The fracture energy *G*_f_ obtained from the experimental results was used as an index to evaluate the bond strength of the FRP–concrete interface. The study analysed the probability distribution type of *G*_f_ and the reliability index *β* of FRP–concrete interface subjected to the dual action of wet–dry cycles in salty water and sustained loading. The results showed that the mean values of *G*_f_ and *β* decreased with increasing wet–dry cycle times. Meanwhile, wet–dry cycles and sustained loading exposure increased the dispersion (COV) of *G*_f_. Five widely used test methods were adopted to verify the possible distribution types of *G*_f_. The best-fitting distribution type was found to be a Lognormal distribution, and neither wet–dry exposure nor sustained loading exposure changed the probability distribution type of *G*_f_. Based on the best-fit distribution of *G*_f_, the *β* of the specimens was then calculated. The changes in the *β* of *G*_f_ with wet–dry exposure were divided into two stages, comprising a fast turn-off and a slow turn-off where the overall trend obeyed a logarithmic function. In cases of dual action of wet–dry cycles and sustained loading, *β* (or failure probability) decreased (or increased) markedly with higher sustained loading level. Neither the mean values nor the standard deviations of the load changed the regulation of the exponential reduction of *β* with wet–dry cycles, but both had a significant influence on the *β* values. This influence was significant for a small number of wet–dry cycles but insignificant for more than 4000 wet–dry cycles.

## Figures and Tables

**Table 1:** Parameters of the specimens.

Specimens	Number of Pieces	Wet–Dry Cycle *N* (*d*)	Sustained Loading *β* (%)	Concrete Strength *f*_cu_ (MPa)	Young’s Modulus *E*_f_ (GPa)	Ultimate Strength *f*_u_ (MPa)	$G_{f}$ (N/mm)	
							Mean	COV
CC-CON	3	0	0	24.8	236	3947	1.436	0.1635
CC-90-0%	3	90	0	32.1	240	3765	1.354	0.2270
CC-180-0%	3	180	0	37.3	240	3614	1.311	0.3083
CC-360-0%	3	360	0	43.5	234	3579	1.248	0.3304
CC-90-30%	3	90	30	32.1	240	3765	1.300	0.2782
CC-180-30%	3	180	30	37.3	240	3614	1.243	0.3167
CC-360-30%	3	360	30	43.5	234	3579	1.183	0.3310
CC-90-60%	3	90	60	32.1	240	3765	1.228	0.2799
CC-180-60%	3	180	60	37.3	240	3614	1.182	0.3165
CC-360-60%	3	360	60	43.5	234	3579	1.025	0.3936

Note: *E*_f_ and *f*_u_ are the Young’s Modulus and ultimate strength of CFRP.

**Table 2:** Test results of probability distribution of *G*_f_.

Test Methods	Normal	Lognormal	Weibull	Gamma
**(a) *G*_f_ for cases not exposed to environment**				
S–W test	0.230	0.173	-	-
K–S test	>0.150	>0.150	-	-
C–M test	>0.250	>0.250	>0.250	-
A–D test	>0.250	>0.250	0.230	-
χ^2^ test	0.123	0.092	0.079	0.073
**(b) *G*_f_ for cases not subjected to sustained loading**				
S–W test	0.358	0.383	-	-
K–S test	>0.150	>0.150	-	>0.500
C–M test	>0.250	0.378	>0.250	>0.250
A–D test	>0.250	0.445	>0.250	>0.500
χ^2^ test	0.110	0.228	0.113	0.204
**(c) *G*_f_ for 30% sustained loading cases**				
S–W test	0.644	0.655	-	-
K–S test	>0.150	>0.150	-	>0.500
C–M test	>0.250	>0.500	>0.250	>0.500
A–D test	>0.250	>0.500	>0.250	>0.500
χ^2^ test	0.423	0.623	0.454	0.617
**(d) *G*_f_ for 60% sustained loading cases**				
S–W test	0.557	0.639	-	-
K–S test	>0.150	>0.150	-	>0.500
C–M test	>0.250	>0.500	>0.250	>0.500
A–D test	>0.250	>0.500	>0.250	>0.500
χ^2^ test	0.472	0.456	0.558	0.512

Note: S–W test was only used for normal distribution, and “-” stands for no results in test calculation.
